# Haplotype-resolved assemblies provide insights into genomic makeup of the oldest grapevine cultivar (Munage) in China

**DOI:** 10.1093/hr/uhaf274

**Published:** 2025-10-20

**Authors:** Haixia Zhong, Xiaoya Shi, Fuchun Zhang, Jin Yao, Xu Wang, Vivek Yadav, Xiaoming Zhou, Shuo Cao, Songlin Zhang, Chuan Zhang, Jiangxia Qiao, Zhongjie Liu, Yingchun Zhang, Yuting Liu, Hao Wang, Hui Xue, Mengyan Zhang, Tianhao Zhang, Wenrui Wang, Ruoyan Zhao, Tianrong Fan, Zhongqi Liu, Jiacui Li, Ruirui Liu, Yongfeng Zhou, Ling Tian, Xinyu Wu, Hua Xiao

**Affiliations:** Institute of Fruits and Vegetables, Xinjiang Uygur Autonomous Region Academy of Agricultural Sciences, Biological Breeding Laboratory, Xinjiang Uygur Autonomous Region Academy of Agricultural Sciences, The State Key Laboratory of Genetic Improvement and Germplasm Innovation of Crop Resistance in Arid Desert Regions (Preparation), Key Laboratory of Genome Research and Genetic Improvement of Xinjiang Characteristic Fruits and Vegetables, Urumqi, China; State Key Laboratory of Tropical Crop Breeding, Tropical Crops Genetic Resources Institute, Chinese Academy of Tropical Agricultural Sciences, Haikou, China; College of Enology, Heyang Viti-Viniculture Station, Ningxia Helan Mountain's East Foothill Wine Experiment and Demonstration Station, Northwest A&F University, Yangling, China; State Key Laboratory of Tropical Crop Breeding, Shenzhen Branch, Guangdong Laboratory of Lingnan Modern Agriculture, Key Laboratory of Synthetic Biology, Ministry of Agriculture and Rural Affairs, Agricultural Genomics Institute at Shenzhen, Chinese Academy of Agricultural Sciences, Shenzhen, China; Institute of Fruits and Vegetables, Xinjiang Uygur Autonomous Region Academy of Agricultural Sciences, Biological Breeding Laboratory, Xinjiang Uygur Autonomous Region Academy of Agricultural Sciences, The State Key Laboratory of Genetic Improvement and Germplasm Innovation of Crop Resistance in Arid Desert Regions (Preparation), Key Laboratory of Genome Research and Genetic Improvement of Xinjiang Characteristic Fruits and Vegetables, Urumqi, China; School of Management, Shenzhen Polytechnic University, Shenzhen, China; State Key Laboratory of Tropical Crop Breeding, Tropical Crops Genetic Resources Institute, Chinese Academy of Tropical Agricultural Sciences, Haikou, China; State Key Laboratory of Tropical Crop Breeding, Shenzhen Branch, Guangdong Laboratory of Lingnan Modern Agriculture, Key Laboratory of Synthetic Biology, Ministry of Agriculture and Rural Affairs, Agricultural Genomics Institute at Shenzhen, Chinese Academy of Agricultural Sciences, Shenzhen, China; Institute of Fruits and Vegetables, Xinjiang Uygur Autonomous Region Academy of Agricultural Sciences, Biological Breeding Laboratory, Xinjiang Uygur Autonomous Region Academy of Agricultural Sciences, The State Key Laboratory of Genetic Improvement and Germplasm Innovation of Crop Resistance in Arid Desert Regions (Preparation), Key Laboratory of Genome Research and Genetic Improvement of Xinjiang Characteristic Fruits and Vegetables, Urumqi, China; Institute of Fruits and Vegetables, Xinjiang Uygur Autonomous Region Academy of Agricultural Sciences, Biological Breeding Laboratory, Xinjiang Uygur Autonomous Region Academy of Agricultural Sciences, The State Key Laboratory of Genetic Improvement and Germplasm Innovation of Crop Resistance in Arid Desert Regions (Preparation), Key Laboratory of Genome Research and Genetic Improvement of Xinjiang Characteristic Fruits and Vegetables, Urumqi, China; State Key Laboratory of Tropical Crop Breeding, Shenzhen Branch, Guangdong Laboratory of Lingnan Modern Agriculture, Key Laboratory of Synthetic Biology, Ministry of Agriculture and Rural Affairs, Agricultural Genomics Institute at Shenzhen, Chinese Academy of Agricultural Sciences, Shenzhen, China; Institute of Fruits and Vegetables, Xinjiang Uygur Autonomous Region Academy of Agricultural Sciences, Biological Breeding Laboratory, Xinjiang Uygur Autonomous Region Academy of Agricultural Sciences, The State Key Laboratory of Genetic Improvement and Germplasm Innovation of Crop Resistance in Arid Desert Regions (Preparation), Key Laboratory of Genome Research and Genetic Improvement of Xinjiang Characteristic Fruits and Vegetables, Urumqi, China; Institute of Fruits and Vegetables, Xinjiang Uygur Autonomous Region Academy of Agricultural Sciences, Biological Breeding Laboratory, Xinjiang Uygur Autonomous Region Academy of Agricultural Sciences, The State Key Laboratory of Genetic Improvement and Germplasm Innovation of Crop Resistance in Arid Desert Regions (Preparation), Key Laboratory of Genome Research and Genetic Improvement of Xinjiang Characteristic Fruits and Vegetables, Urumqi, China; Institute of Fruits and Vegetables, Xinjiang Uygur Autonomous Region Academy of Agricultural Sciences, Biological Breeding Laboratory, Xinjiang Uygur Autonomous Region Academy of Agricultural Sciences, The State Key Laboratory of Genetic Improvement and Germplasm Innovation of Crop Resistance in Arid Desert Regions (Preparation), Key Laboratory of Genome Research and Genetic Improvement of Xinjiang Characteristic Fruits and Vegetables, Urumqi, China; State Key Laboratory of Tropical Crop Breeding, Shenzhen Branch, Guangdong Laboratory of Lingnan Modern Agriculture, Key Laboratory of Synthetic Biology, Ministry of Agriculture and Rural Affairs, Agricultural Genomics Institute at Shenzhen, Chinese Academy of Agricultural Sciences, Shenzhen, China; State Key Laboratory of Tropical Crop Breeding, Shenzhen Branch, Guangdong Laboratory of Lingnan Modern Agriculture, Key Laboratory of Synthetic Biology, Ministry of Agriculture and Rural Affairs, Agricultural Genomics Institute at Shenzhen, Chinese Academy of Agricultural Sciences, Shenzhen, China; State Key Laboratory of Tropical Crop Breeding, Tropical Crops Genetic Resources Institute, Chinese Academy of Tropical Agricultural Sciences, Haikou, China; State Key Laboratory of Tropical Crop Breeding, Shenzhen Branch, Guangdong Laboratory of Lingnan Modern Agriculture, Key Laboratory of Synthetic Biology, Ministry of Agriculture and Rural Affairs, Agricultural Genomics Institute at Shenzhen, Chinese Academy of Agricultural Sciences, Shenzhen, China; Institute of Fruits and Vegetables, Xinjiang Uygur Autonomous Region Academy of Agricultural Sciences, Biological Breeding Laboratory, Xinjiang Uygur Autonomous Region Academy of Agricultural Sciences, The State Key Laboratory of Genetic Improvement and Germplasm Innovation of Crop Resistance in Arid Desert Regions (Preparation), Key Laboratory of Genome Research and Genetic Improvement of Xinjiang Characteristic Fruits and Vegetables, Urumqi, China; State Key Laboratory of Tropical Crop Breeding, Shenzhen Branch, Guangdong Laboratory of Lingnan Modern Agriculture, Key Laboratory of Synthetic Biology, Ministry of Agriculture and Rural Affairs, Agricultural Genomics Institute at Shenzhen, Chinese Academy of Agricultural Sciences, Shenzhen, China; State Key Laboratory of Tropical Crop Breeding, Tropical Crops Genetic Resources Institute, Chinese Academy of Tropical Agricultural Sciences, Haikou, China; State Key Laboratory of Tropical Crop Breeding, Shenzhen Branch, Guangdong Laboratory of Lingnan Modern Agriculture, Key Laboratory of Synthetic Biology, Ministry of Agriculture and Rural Affairs, Agricultural Genomics Institute at Shenzhen, Chinese Academy of Agricultural Sciences, Shenzhen, China; State Key Laboratory of Tropical Crop Breeding, Tropical Crops Genetic Resources Institute, Chinese Academy of Tropical Agricultural Sciences, Haikou, China; State Key Laboratory of Tropical Crop Breeding, Shenzhen Branch, Guangdong Laboratory of Lingnan Modern Agriculture, Key Laboratory of Synthetic Biology, Ministry of Agriculture and Rural Affairs, Agricultural Genomics Institute at Shenzhen, Chinese Academy of Agricultural Sciences, Shenzhen, China; State Key Laboratory of Tropical Crop Breeding, Tropical Crops Genetic Resources Institute, Chinese Academy of Tropical Agricultural Sciences, Haikou, China; College of Enology, Heyang Viti-Viniculture Station, Ningxia Helan Mountain's East Foothill Wine Experiment and Demonstration Station, Northwest A&F University, Yangling, China; State Key Laboratory of Tropical Crop Breeding, Tropical Crops Genetic Resources Institute, Chinese Academy of Tropical Agricultural Sciences, Haikou, China; State Key Laboratory of Tropical Crop Breeding, Tropical Crops Genetic Resources Institute, Chinese Academy of Tropical Agricultural Sciences, Haikou, China; State Key Laboratory of Tropical Crop Breeding, Tropical Crops Genetic Resources Institute, Chinese Academy of Tropical Agricultural Sciences, Haikou, China; State Key Laboratory of Tropical Crop Breeding, Tropical Crops Genetic Resources Institute, Chinese Academy of Tropical Agricultural Sciences, Haikou, China; Gansu Key Laboratory of Conservation and Utilization of Biological Resources and Ecological Restoration in Longdong, School of Agriculture and Bioengineering, Longdong University, Qingyang, China; State Key Laboratory of Tropical Crop Breeding, Tropical Crops Genetic Resources Institute, Chinese Academy of Tropical Agricultural Sciences, Haikou, China; State Key Laboratory of Tropical Crop Breeding, Shenzhen Branch, Guangdong Laboratory of Lingnan Modern Agriculture, Key Laboratory of Synthetic Biology, Ministry of Agriculture and Rural Affairs, Agricultural Genomics Institute at Shenzhen, Chinese Academy of Agricultural Sciences, Shenzhen, China; School of Management, Shenzhen Polytechnic University, Shenzhen, China; Institute of Fruits and Vegetables, Xinjiang Uygur Autonomous Region Academy of Agricultural Sciences, Biological Breeding Laboratory, Xinjiang Uygur Autonomous Region Academy of Agricultural Sciences, The State Key Laboratory of Genetic Improvement and Germplasm Innovation of Crop Resistance in Arid Desert Regions (Preparation), Key Laboratory of Genome Research and Genetic Improvement of Xinjiang Characteristic Fruits and Vegetables, Urumqi, China; Institute of Fruits and Vegetables, Xinjiang Uygur Autonomous Region Academy of Agricultural Sciences, Biological Breeding Laboratory, Xinjiang Uygur Autonomous Region Academy of Agricultural Sciences, The State Key Laboratory of Genetic Improvement and Germplasm Innovation of Crop Resistance in Arid Desert Regions (Preparation), Key Laboratory of Genome Research and Genetic Improvement of Xinjiang Characteristic Fruits and Vegetables, Urumqi, China; State Key Laboratory of Tropical Crop Breeding, Shenzhen Branch, Guangdong Laboratory of Lingnan Modern Agriculture, Key Laboratory of Synthetic Biology, Ministry of Agriculture and Rural Affairs, Agricultural Genomics Institute at Shenzhen, Chinese Academy of Agricultural Sciences, Shenzhen, China

## Abstract

Munage, an ancient grape variety that has been cultivated for thousands of years in Xinjiang, China, is renowned for its exceptional fruit traits. There are two main types of Munage: white fruit (WM) and red fruit (RM). However, the lack of a high-quality genomic resources has impeded effective breeding and restricted the potential for expanding these varieties to other growing regions. In this study, we assembled haplotype-resolved genome assemblies for WM and RM, alongside integrated whole genome resequencing (WGS) data and transcriptome data to illuminate the origin, private mutations and selection in Munage. Our analyses suggest that Munage likely shares a common ancestor with Eurasian grapes that originated in West Asia. Selective analysis between Munage clones and Eurasian grapes mapped genomic signals of selection in Munage grapes, with genes enriched in processes including cell maturation, plant epidermal cell differentiation, and root epidermal cell differentiation. We also identified 283 somatic mutation sites between WM and RM, along with differential selection on genome and expressed genes. These findings provide crucial genetic resources for investigating the genetics of the ancient Chinese grape variety, Munage, and will facilitate the genetic improvement in grapevine using this ancient cultivar as a gene donor.

## Introduction

The cultivated grapevine (*Vitis vinifera* L.) is closely associated with human civilization. Its extensive genetic diversity has enabled its use for a wide range of purposes, while the grapevine has also come to symbolize cultural identity in many regions due to its central role in culinary and social practices [1, 2]. The Eurasian grapes were initially domesticated and cultivated in a region extending from the eastern Mediterranean coast to the Caucasus, situated between the Caspian and Black Seas [3–7], and were subsequently introduced to Europe, Asia, and other regions [8]. There is no naturally occurring Eurasian grape in China. Historical records indicate that around 100 BC, grapes were introduced to the ancient Chinese capitals via the Silk Road [9, 10]. However, grape remains unearthed from the Yanghai cemetery show that grapes had already been brought into Xinjiang 2300 years ago, which is 200 years earlier than the opening of the ancient Silk Road [11–13].

Xinjiang has a long history of grape cultivation and has thus inherited a rich variety of ancient native grape germplasm resources with irreplaceable genes [14, 15]. Traditional native varieties include Thompson Seedless, Mare’s Teat (also known as Milk), and Munage, among others. As one of the most representative and high-quality cultivars in Xinjiang [16, 17], Munage not only possesses a unique flavor but is also highly favored by consumers for its large berries, delicate skin, and juicy flesh, and is thus widely cultivated [18]. Throughout thousands of years of extensive selection and cultivation, many variations have accumulated, including both beneficial and deleterious ones. These variations in germplasm resources are invaluable for breeding and the development of new varieties. However, the trait-specific genes underlying these characteristics have yet to be systematically investigated and utilized.

Munage can be classified into two types based on skin color: red Munage (RM) and white Munage (WM). This ancient variety exhibits unique regional adaptability and stress resistance [19, 20], and its favorable traits and specific genetic resources warrant further exploration and utilization. In addition, it is widely believed that RM grapes originated as a bud mutation of WM, although clear scientific evidence is currently lacking. Bud sport mutation is a common phenomenon in fruit plants and plays a vital role in improving the overall quality of grapevines. For instance, a famous Chinese variety Jinzao Wuhe is a bud sport of Himrod Seedless, characterized by enlarged berrie [21]. Moreover, Thompson Seedless is also considered to be a shoot mutant of Sultanina, which was further distributed and maintained by cuttings [22]. Other bud sports that have been identified and successfully applied in grapevine breeding include early-ripening cultivars, such as ‘Fengzao’, ‘Tiangong Moyu’, and ‘Nantaihutezao’ [23–25], as well as berry skin color mutants such as ‘Pinot Grigio’, ‘Pinot Blanc’, and ‘Benitaka’ [26–28]. The variants observed between WM and RM grapes will provide a valuable opportunity to understand the effects of somatic mutations on grape traits. More recently, genome sequencing in grapes has greatly contributed to our understanding of the structure of the *Vitis* genome [22]. In the past decade, the utility of haplotype assemblies for identifying genes associated with agronomic traits has been well demonstrated in grapevine [22, 29–31].

In this study, we generated high-quality haplotype-resolved genome assemblies of the ancient Chinese grape variety Munage. Comparative genomics was employed to identify selected differential segments and structural variations (SVs) in the genomes of different varietal types of the ancient grape variety Munage. Combining the whole genome sequencing data of Munage and various Eurasian grapes allows us to elucidate the evolutionary relationships and explore the sweep selection on the genome of this ancient grape variety. Furthermore, we analyzed transcriptome data from multiple Munage tissues to identify differentially expressed genes associated with key traits, particularly those related to fruit color. This work improves our understanding of the origin and domestication history of Munage, provides valuable genomic resources for future grape genome research, and offers a foundation for developing new grape germplasm and advancing breeding strategies.

## Results

### Haplotype-resolved assemblies of Munage genome

**Figure 1:**
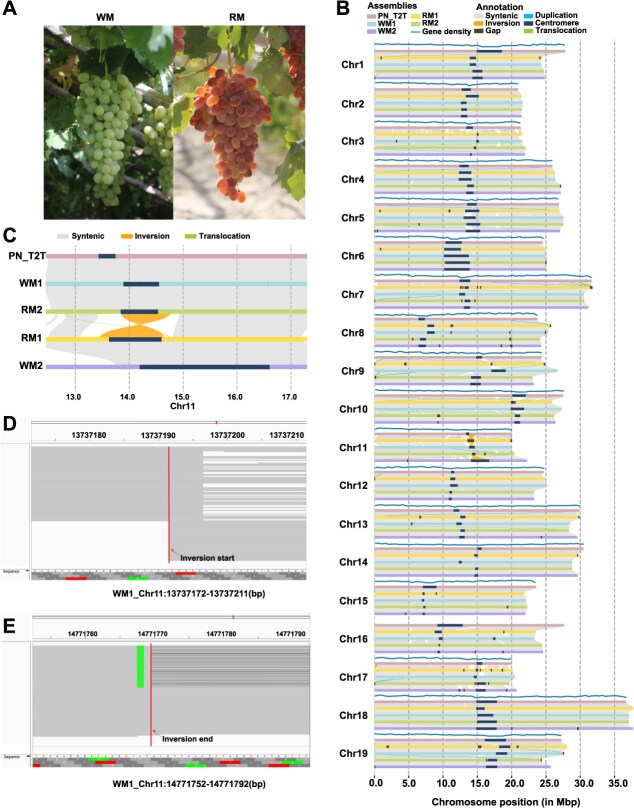
Haplotype-resolved assemblies of the RM and WM genomes. (A) Fruit images of RM and WM. (B) Collinearity alignments between the PN_T2T genome and the WM1, WM2, RM1, and RM2 genomes. (C) Schematic diagram of structural variations in the 13–15 Mb region of chromosome 11. (D-E) IGV visualization for inversion verification. The IGV screenshots show the inverted breakpoints of RM1, with the red vertical line indicating the locations of the breakpoints.

The plant materials used in this study consisted of an 80-year-old clonally propagated individual of white Munage (WM) from Xinjiang, China, and a red Munage (RM) individual derived from a mother tree over 30 years old (Fig. 1A). To characterize WM and RM at the genomic level, we performed PacBio sequencing on both varieties, generating 64.27 Gb of HiFi reads for WM and 49.78 Gb of HiFi reads for RM. Hi-C data were generated using the Illumina HiSeq X Ten platform, yielding 52.35 Gb of Hi-C reads for WM and 52.67 Gb of Hi-C reads for RM.

The genome sizes of the two species, determined using the k-mer metric method with HiFi reads, indicated a genome size of ~450 974 828 bp for WM and ~461 596 620 bp for RM (Fig. S1A, Table S1). Then, we performed an initial assembly using HiFiasm, which yielded four haplotypes for the two species at the contig level. The contig N50 values were 24.32 Mb for haplotype 1 of WM (WM1), 26.33 Mb for haplotype 2 of WM (WM2), 15.55 Mb for haplotype 1 of RM (RM1), and 17.08 Mb for haplotype 2 of RM (RM2) (Tables S1 and S2).

Through Hi-C data integration, we obtained haplotype-resolved sequence assemblies for white and red Munage. The sizes of the assemblies were 488 103 118 bp for WM1, 489 354 377 bp for WM2, 489 516 681 bp for RM1, and 480 619 624 bp for RM2 (Table S2). The BUSCO completeness scores were 98.3%, 98.6%, 98.3%, and 98.5%, respectively, indicating high assembly quality (Fig. S1B). There is a highly evident collinearity across the entire genome of WM, RM, and PN_T2T (Fig. 1B), but we also we identified some large variations among haplotypes, e.g. a large inversion in the 13- to 15-Mb region of chromosome 11 between two haplotypes of both RM and WM (Fig. 1C–E).

### Comparative genomic analysis

**Figure 2:**
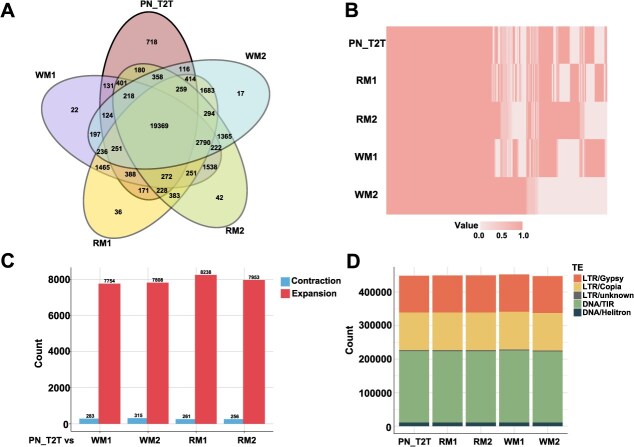
Comparisons among the PN_T2T, RM, and WM Genomes. (A) The number of shared and unique gene families among the PN_T2T, WM1, WM2, RM1, and RM2 genome assemblies. (B) Presence and absence of gene families in the PN_T2T, RM1, RM2, WM1, and WM2 genomes. The pink gradient indicates the abundance of each gene family when present in the genome, with the lightest color representing the absence of the gene family in that genome. (C) The numbers of contracted and expanded gene families in WM1, WM2, RM1, and RM2 compared to PN_T2T. (D) The number of major TE types across the whole genomes of WM1, WM2, RM1, RM2, and PN_T2T.

Although strong collinearity among the five genomes—WM1, WM2, RM1, RM2, and PN_T2T [32] was observed (Fig. 1B), certain differences were detected in gene composition (Fig. 2A and B). Gene annotation identified 33 942, 34 034, 35 292, and 35 007 genes in WM1, WM2, RM1, and RM2, respectively (Table S2). By comparing the gene annotations among the five haplotypes, 718 orthologous genes unique to PN_T2T were identified (Fig. 2A). These genes were mainly enriched in pathways related to secondary metabolism, defense response, asymmetric cell division, and secondary metabolite biosynthesis during embryonic development (Table S3, Fig. S2A). Further, we identified 22 unique orthologous genes in WM1, which were mainly enriched in pathways such as response to stimulus, response to external biotic stimulus, response to biotic stimulus, biological processes involved in interspecies interactions, and obsolete multi-organism processes (Fig. 2A, Fig. S2B, Table S4). There are 17 unique genes in WM2, but no GO enrichment pathway was identified. The functional annotation of these genes is primarily associated with RNA-mediated transposition, ribonuclease activity, or kinase activity (Table S5). RM1 contained 36 unique orthologous genes, which were primarily enriched in pathways such as obsolete cellular nitrogen compound metabolic processes, obsolete RNA polyadenylation, obsolete nitrogen compound metabolic processes, RNA 3′-end processing, and RNA metabolic processes (Fig. 2A, Table S6, Fig. S2C). RM2 has 42 unique orthologous genes, which are mainly enriched in pathways including plant organ development, RNA splicing via transesterification reactions with bulged adenosine as a nucleophile, RNA splicing via transesterification reactions, and RNA splicing (Fig. 2A, Fig. S2D, Table S7). Compared with PN_T2T, the numbers of expanded gene families in WM1, WM2, RM1, and RM2 were 7754, 7808, 8238, and 7953, respectively, while the numbers of contracted gene family were 283, 315, 261, and 256, respectively (Fig. 2C). We also conducted statistics on transposable element (TE) annotations, with WM1, WM2, RM1, and RM2 showing proportions of 67.04%, 67.15%, 67.02%, and 66.56% of the whole genome, respectively. Additionally, the proportions of different TE types among the five haplotypes were similar, with TIR being the major TE (~23%) (Fig. 2D).

### Population subdivision

**Figure 3:**
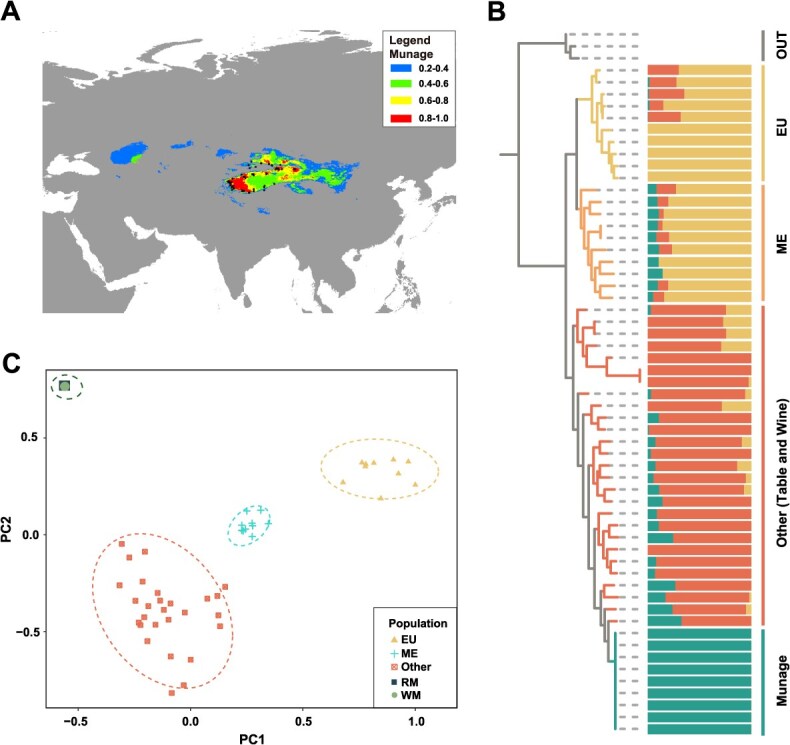
Population sub-division of Munage grapes. (A) Predicted distribution area of Munage, with black dots indicating the primary planting regions. The color from blue to red means the possibility from lower to higher. The color gradient from blue to red indicates the likelihood of suitability, ranging from lower to higher. (B) Phylogenetic tree with admixture analysis, where branches represent different group. The admixture plot shows *K* = 4. (C) A PCA supporting the designation of four groups. EU: European wild grapes; ME: Wild grapes from the Middle East and Caucasus region; Other: 20 Eurasia cultivated grapes; Munage: white Munage and red Munage grapes; OUT: *Vitis californica*.

Munage is a locally cultivated variety widely grown in Xinjiang. Predictions based on climate and sampling locations indicate that its most suitable growing regions are in northwestern China (Fig. 3A). There are no naturally occurring Eurasian grape species in China, and it is widely believed that the ancestors of Xinjiang's local grape varieties, including Munage, originated from West Asia, the domestication center of Eurasian cultivated grapes. To investigate the relationship of Munage with other Eurasian grapes, we analyzed resequencing data from 59 grape accessions. These included 10 wild grapes (*V. vinifera* ssp. *sylvestris*) from Europe (EU), 10 wild grapes (*V. vinifera* ssp. *sylvestris*) from the Middle East and Caucasus region (ME), 20 cultivated grapes (*V. vinifera* ssp. *Vinifera*, also known as Eurasia grapes), and 9 Munage grapes from Xinjiang (Table S8), together with three *Vitis californica* samples from North America serving as the outgroup (OUT) (Table S8).

The phylogenetic tree analysis using FastTree software, with PN_T2T as the reference genome, revealed that wild and cultivated grapes form two distinct clades, reflecting an early divergence between the two groups (Fig. 3B). The 20 domesticated grape varieties, including those used for fresh eating (Table) and wine making (Wine), along with Munage, formed a well-supported monophyletic cluster. Among these, the cultivar most closely related to Munage was ISRAEL-U.I, a table grape originating from Israel (Fig. S3A) [4]. This finding suggests that Munage shares a common origin with domesticated Eurasian grapes. Principal component analysis (PCA) further confirmed the genetic differentiation among these populations. At the PC1 level, Munage and other Eurasian cultivated grapes are positioned on the left, in contrast to wild grapes on the right. However, at the PC2 level, Munage and other Eurasian cultivated grapes are separated (Fig. 3C), indicating that the Munage genome experienced different early selection pressures compared to other cultivated grapes during domestication.

### Selective sweep in the Munage grape

**Figure 4:**
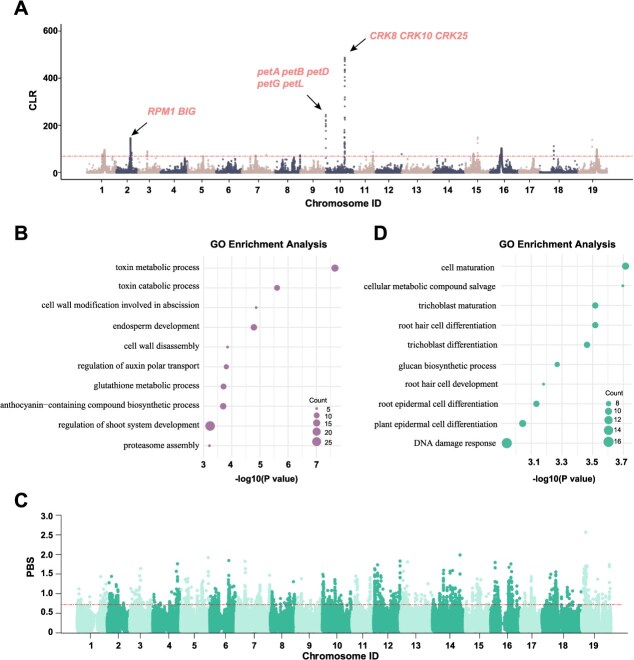
Selective sweep in the Munage grape. (A) SweeD analyses of the Munage group. (B) GO term enrichment of the biological process in the top 1% of genes in the SweeD analyses. (C) PBS analysis based on SNP variant information, using the Munage, Other, and ME groups. (D) GO term enrichment of the biological process in the top 1% of genes in the PBS analyses.

To identify the selective sweep region in Munage, we performed composite likelihood ratio (CLR) analysis on nine Munage grapevine samples using SweeD software. Several selected genomic regions were detected, particularly on chromosomes 2 and 10 (Fig. 4A). On chromosome 2, the *RPM1* gene [33] was located at the selection region. *RPM1* is a classical plant resistance gene (R gene) that encodes a protein capable of recognizing pathogen effectors and activates the plant immune system to defend against pathogen invasion. Another identified gene is *BIG* [34], which participates in the regulation of plant hormone signaling, including auxin and gibberellin pathways. *BIG* modulates cellular sensitivity and response to these hormones, thereby influencing plant growth and development. On chromosome 10, a gene cluster comprising *petA, petB, petD, petG*, and *petL* [35] was identified; these genes encode proteins associated with the cytochrome b6f complex of the photosynthetic electron transport chain, playing essential roles in photosynthesis. In addition, *CRK8, CRK1,* and *CRK25* [36] were detected; these cysteine-rich receptor-like kinases (CRKs) are involved in plant immune responses to various pathogens and environmental stresses. We further performed GO enrichment analysis on the top 1% of genomic regions with the highest composite likelihood ratios. These regions were predominantly enriched in pathways including camalexin metabolic process, regulation of shoot system development, anthocyanin-containing compound biosynthetic process, glutathione metabolic process, regulation of auxin polar transport, cell wall disassembly, endosperm development, cell wall modification involved in abscission, toxin catabolic process, and proteasome assembly (Fig. 4B, Table S9). These pathways are closely associated with the regulation of normal physiological activities in plants.

We also performed Population Branching Structure (PBS) analysis to identify differential divergent selective regions between Munage and other cultivated grapes, revealing significant differentiation across numerous regions distributed across 19 chromosomes (Fig. 4C). We selected the top 1% of regions with the highest PBS values for gene ontology (GO) enrichment analysis. These regions were primarily enriched in pathways, such as cell maturation, plant epidermal cell differentiation, root epidermal cell differentiation, root hair cell development, glucan biosynthetic process, trichoblast differentiation, root hair cell differentiation, trichoblast maturation, cellular metabolic processes, and compound salvage (Fig. 4D, Table S10). These pathways associated with essential physiological processes and the maintenance of vital activities in plants, and are likely linked to adaptation to the specific environment in Xinjiang.

### The variant and differential expression genes in grapes

Although RM and WM are genetically identical clones (IBD, 0.98; Fig. S3B), there are significant differences between them. WM features green shoots with a slight brownish tint and produces crispy, sweet fruit flesh, whereas RM shows green shoots with a reddish-purple hue, reddish branches, and crispy, slightly tart fruit flesh. Several distinct selection signals were observed between WM and RM (Figs S4 and S5, Table S11). Genes located within the top 1% of the selected regions in RM were enriched in pathways related to anthocyanin-containing compound biosynthesis, positive regulation of anthocyanin biosynthesis, positive regulation of anthocyanin metabolism, regulation of anthocyanin biosynthesis, and pigment biosynthesis. These processes show a strong association with berry color (Fig. S5B, Table S12).

**Figure 5:**
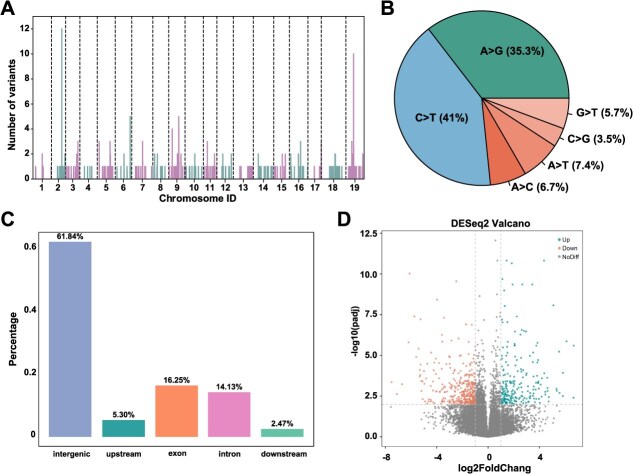
Variants and Differential Selection between RM and WM. (A) The number of somatic variants identified on each chromosome between WM and RM. (B) The proportion of somatic variant types present in WM and RM. The two transition types are shown in different shades of teal, while the four transversion types are shown in different shades of orange. (C) The percentage of somatic variants distributed at different positions within genes. (D) Differential gene expression analysis based on WM and RM transcriptome data.

To further explore the genetic differences between WM and RM, we examined sites that differed between the two cultivars but were consistent within each group. A total of 283 somatic mutation sites were identified. They are distributed throughout the entire genome, with slightly higher densities observed on chromosomes 2, 5, 9, and 19 (Fig. 5A). Among these, the two transition types, C > T (41%) and A > G (35.3%), were more prevalent than the four transversion types, each representing less than 10% of the variants (Fig. 5B). The majority of variants (61.8%) were located in intergenic regions, 30.4% were within genes (16.3% in exons and 14.1% in introns), and only 7.8% were situated in gene flanking regions (2 kb upstream and downstream) (Fig. 5C).

We also integrated RNA-seq data from RM and WM peels for temporal analysis of differentially expressed genes. A total of 578 differentially expressed genes were identified, including 216 up-regulated genes and 362 down-regulated genes in RM relative to WM (Fig. 5D). The up-regulated genes were primarily enriched in pathways related to water transport, fluid transport, transmembrane transport, and solute transport (Fig. S6A, Table S13), as well as related to skin color, including pigment metabolism and biosynthesis. Conversely, down-regulated genes were enriched in pathways involved in heat response, temperature stimulus response, pigment metabolism, and pigment biosynthesis processes (Fig. S6B, Table S14). These findings suggest that somatic mutations between WM and RM may influence gene function or expression in Munage grapes.

## Discussion

In this study, we provide valuable resources for a deeper understanding of the genetic basis, domestication history, and breeding of Munage grapes through the construction of high-quality genome assemblies and population genetic analyses. Our results indicate that Munage shares a common ancestor with Eurasian cultivated grapes originating in West Asia; however, through long-term artificial selection, it has undergone genomic adaptation to the climatic conditions of Xinjiang and genetically diverged from other Eurasian grape varieties. Although clonal propagation preserves the stability of favorable phenotypes and genotypes, the accumulation of somatic mutations has probably led to phenotypic variation between white Munage (WM) and red Munage (RM).

Genomes serve as indispensable resources for both basic research and breeding, as they provide comprehensive insights into genetic variations and thereby enable the development of improved traits and optimized crop varieties [37–41]. In 2023, the first telomere-to-telomere grape genome assembly was published using PN40024 [32], marking a major advance in grapevine genomics. This complete assembly has substantially enhanced our understanding of grape genetics and has improved the precision of breeding efforts. However, most cultivated grapevines are highly heterozygous [42–46], which poses a challenge in studying their genomes. With the advent of chromium sequencing technology and high-throughput chromatin conformation capture (Hi-C) [47], an increasing number of high-quality plant genomes have been assembled, making diploid genome assembly and annotation a routine practice. Nonetheless, for ancient grape germplasm resources, phylogenetic studies of Eurasian species of native grapes are even more challenging to carry out due to the lack of high-quality reference genome sequences and genome annotation information. Thus, we generated haplotype-resolved high-quality genomes for this renowned cultivar; this resource will not only facilitate in-depth research on the cultivar itself but also provide critical genomic data support for studies on its closely related varieties, genetic improvement, and the evolutionary mechanisms of the entire grapes. The estimated genome size of *V. vinifera* is ~500 Mb ^48^ and our results indicate that the genome sizes of white Munage (WM) and red Munage (RM) range from 480 to 490 Mb, respectively in line with the recently published grape assemblies. Both genomes exhibited a heterozygosity level of 1.5%, reflecting the high degree of heterozygosity characteristic of grapevines, similar to cultivars such as Chasselas and Ugni Blanc [48]. A high BUSCO score (> 98.3) across all Munage haplotypes indicates that the genome assemblies are of high quality, with accurate annotations and minimal fragmentation or duplication. We annotated 33 942 (WM1), 34 034 (WM2), 35 292 (RM1), and 35 007 (RM2) genes, with TE annotations ranging between 67.15% and 66.56%. The annotated genes and TE annotations in previous grape assemblies, including Shanputao (*V. amurensis*), Noble (*M. rotundifolia*), PN40024 v2, and Sultanina v2, were lower than those in the current study, highlighting the importance of our research in terms of assembly quality [49].

According to historical records, the grapes domesticated in Xinjiang were introduced from Central Asia; however, there is a lack of genomic evidence, and the origin of Munage remains unclear. Based on population genetic analyses, we found that Munage clusters with Eurasian cultivated grapes and is closely related to U.I, a table grape originating from Israel [4]. According to Dong *et al.*, U.I belongs to group CG1 (Fig. S3A), which comprises table grape varieties from the West Asia region. Grapes within this group have a long domestication history, dating back ~11 000 years. Therefore, Munage likely shares common ancestry with the CG1 grapes that originated in West Asia, and its ancestors were subsequently introduced to Xinjiang via Central Asia. (Fig. 3B) [11, 50].

Although the ancestor of Munage originated in West Asia, its most suitable cultivation region has shifted to Northwest China, particularly Xinjiang (Fig. 3A). This indicates that adaptive selection occurred during the domestication process. The distinct selective sweeps observed are also supported by PCA and PBS analyses, highlighting the genomic differences between Munage and other cultivated grapes. (Figs 3C and 4C). The significant differences in selective regions can be observed across 19 chromosomes (Fig. 4C). During domestication, selection has been primarily driven by traits favored by humans [51], leading to the continuous accumulation of genetic variants that enhance these desirable traits and, over time, causing the genetic composition of domesticated species to diverge from that of their wild relatives, thereby reflecting the selective pressures imposed during domestication [52–54]. In this study, the PBS analysis with multiple populations showed some selective sweep regions in the chromosomes. Significant differentiation across the chromosome region in the population provides strong evidence of natural selection over time in Munage.

Clonal propagation is widely used in grape production, providing significant advantages in maintaining the stability of favorable phenotypes and genotypes [11, 55–57]; however, the clonal process may also accumulate a large number of heterozygous somatic variations [58]. In this context, once beneficial mutations are identified during grape breeding, breeders can achieve rapid selection through clonal propagation [46, 59]. Recent genomic studies, such as those on ‘Yan73’ provided insights into the color development mechanism in grapevines [30]. White berries are assumed to have emerged from independent mutations [60]. It is assumed that RM originated by the bud mutation in WM, but distinct traits are present among them, especially in fruit peel color. In our findings, we provided scientific evidence to their clonal relationship through IBD analysis (0.98 between WM and RM). We hypothesize that certain somatic variants between WM and RM may underlie their phenotypic difference. To investigate this, we identified the somatic mutations between WM and RM by identifying specific variants in each group, and a total of 283 somatic mutation sites were identified between WM and RM. These variations were distributed across the genome, with several peaks observed, including a notable cluster on chromosome 2, a region previously reported to be associated with berry color (Fig. 5A) [43]. Only 30% of these variations are located on genes, indicating that these variations probably mainly affect gene expression rather than altering the genes themselves (such as inactivation or functional changes). As expected, a total of 578 differentially expressed genes were identified based on RNA-seq data from RM and WM peels. (Fig. 5D). Both the up-regulated genes and down-regulated genes were enriched in pathways related to skin color, including pigment metabolism and biosynthesis (Fig. S6, Tables S13 and S14). However, our recent work relies on bioinformatics approaches and transcriptomic data, necessitating follow-up experiments to validate these findings and identify the causal genes for color change in the future. These experiments include quantitative real-time polymerase chain reaction (qRT-PCR) to verify the expression levels of the candidate genes and performing functional studies, such as gene knockouts or overexpression, to confirm the roles of these candidate genes in the observed phenotype.

In summary, our findings not only provide high-quality genomic resources for the Munage grapes but also reveal the roles of clonal propagation, selective pressures, and somatic mutations in shaping fruit phenotypic variation. These results further offer theoretical guidance and data support for future genetic improvement of grapes and functional gene studies.

## Materials and methods

### Plant materials and sampling

An ancient grape variety with specific traits, a standard mother plant of red Munage over 30 years old, and a standard mother plant of white Munage over 80 years old were selected from multiple locations in Xinjiang, China. High-quality DNA from leaves to generate haplotype-resolved genome assemblies. To achieve this, we employed PacBio whole-genome high-fidelity (HiFi) sequencing at 100× coverage, high-throughput chromosome conformation capture (Hi-C) proximity ligation, and Illumina short-read sequencing. Genome annotation was performed using Illumina RNA-seq data from various Munage tissues. Details of the genetic resources used are provided in Table S8.

### DNA sequencing and library preparation

The CTAB method was used to extract high-quality genomic DNA from leaf tissue samples. The concentration and purity of the DNA were assessed using a Qubit fluorometer from Thermo Fisher Scientific. Gel electrophoresis was performed to verify DNA integrity. To sequence the DNA of the standard mother plant of the Xinjiang native ancient grape variety Munage, third generation of PacBio Sequel CCS 100× with HiFi data volume of up to 50Gb and two cells were used. For PacBio HiFi sequencing, a standard SMRTbell library was constructed using the SMRTbell Express Template Prep Kit 2.0, following the manufacturer's recommendations (Pacific Biosciences, CA, USA). Additionally, the Munage genome haplotypes were assembled using second-generation 50× Hi-C combination sequencing technology.

### Total RNA extraction, library preparation and sequencing

To perform RNA sequencing, we utilized the NEBNext® Ultra™ II Directional RNA Library Prep Kit for Illumina® (New England Biolabs, MA, USA) to extract total RNA from various plant parts, including the leaves, pulp, stalk, and root of the Munage grapevine. Subsequently, paired end reads of 150 base pairs were generated on the Illumina NovaSeq platform. The sample details are presented in Table S8.

### De novo haplotype-resolved genome assembly and quality assessment

We first used the Hi-C Integrated Assembly mode of HiFiasm, combining Munage HiFi and Hi-C data, to generate two contig-level haplotype genomes. Genomic heterozygosity was estimated using GenomeScope (v2.0) [61], applying a k-mer-based strategy applied to the raw HiFi reads. Next, RagTag was used with the Cabernet Sauvignon genome as a reference to infer the approximate order of contigs along the chromosomes. Hi-C sequencing data were then utilized to anchor all contigs using Juicer (v1.5) [62]. The genome structure was further refined with 3D-DNA, and manual adjustments were performed in Juicebox (v1.11.08, https://github.com/aidenlab/Juicebox), followed by a secondary application 3D-DNA [63] to obtain scaffold-level genome. Genome quality was assessed using BUSCO [64] with the embryophyta_odb10 database to evaluate the genome’s completeness.

### Annotation of genes and transposable elements

We employed a comprehensive genome-wide annotation pipeline (https://github.com/unavailable-2374/Genome-Wide-Annotation-Pipeline) annotate the Munage genome, utilizing RNA-seq data from multiple tissues. Initially, RNA sequences were aligned to repeat-masked assemblies with Hisat2 (v2.10.2), followed by transcript assembly with StringTie (v1.3.0) [65]. Transcripts and UniProt (https://www.uniprot.org/) were used as supporting evidence for gene identification. Preliminary gene models were constructed using tools such as exonerate, genewise, and transfrag. Following the initial gene models construction, AUGUSTUS (v3.4.0) [66] was applied to refine predictions. Genes located in duplicated regions, with CDS regions shorter than 90 bases, with coding sequences shorter than 90 bp, or lacking supporting evidence were filtered out. Missing genes were supplemented where possible, and alternative splicing analysis was performed on the remaining intact genes. Final gene models were verified using a hidden Markov model derived from the Pfam database.

For transposable element (TE) annotation, a TE library was first constructed using RepeatModeler (open-2.0.3) with the -LTRStruct option [67]. Genome-wide TE annotation was then conducted using RepeatMasker (open-4.1.2, https://github.com/rmhubley/RepeatMasker) with the -e rmblast, −lcambig, and slow models.

### Genome comparison between haplotypes and reference genome

For comparative genomic analysis, the WM1, WM2, RM1, RM2, and PN_T2T genomes were first aligned using Minimap2 (v.2.21) [68], and the resulting BAM files were indexed with SAMtools (v1.4) [69]. Synteny relationships and structural rearrangements among the genomes were subsequently identified using SyRI (v.1.7.0) [70]. The alignment results were finally visualized with plotsr [71].

### SNP calling and filtering

We utilized resequencing data from a total of 59 grape accessions, comprising 10 wild grapes (*Vitis vinifera* subsp. *sylvestris*) from Europe (EU), 10 wild grapes from the Middle East (ME), 27 cultivated grapes (Other), three white Munage, and six red Munage. Additionally, three Muscadine grape samples were included as an outgroup. Of these, nine Munage accessions were newly resequenced in this study, while the remaining datasets were retrieved from the National Center for Biotechnology Information (NCBI). Quality control of the resequencing data was performed using fastp (v0.21) [72] with default parameters. Subsequently, the quality-filtered paired-end data were aligned to the PN_T2T complete reference genome, and SNPs were identified across the 59 resequenced samples using GTX (v2.2.1). To reduce false-positive variant calls, we applied stringent filtering with VCFtools [73]. Specifically, we removed genotypes with a genotype quality less than 20 (option-minGQ 20), SNPs with more than two alleles (options -min-alleles 2--max-alleles 2), and SNPs with more than 20% missing genotypes (option -max-missing 0.8). Indels were also filtered by VCFtools using command —remove-indels.

### Gene ontology enrichment analysis

We first converted the genome into protein sequences based on the gene annotation file, and then annotated the protein sequences using the eggNOG-mapper web server (eggNOG v5.0) [74]. Each protein sequence under analysis was ultimately assigned a functional description corresponding to its ortholog. The final annotation results were used as the background, and GO enrichment analysis of the target genes was performed using TBtools [75].

### Phylogeny and population structure

Principal component analysis (PCA) was performed using PLINK [76]. Phylogenetic trees were constructed using FastTree (v2.1.10) [77] based on GTR models. Population structure was inferred using Admixture (v1.3.0) [78] with K values ranging from 2 to 10, based on nuclear SNPs. IBD was calculated using PLINK with parameter: —genome.

### Selective sweep

To detect signatures of selective sweeps in Munage populations, we applied the Composite Likelihood Ratio (CLR) method implemented in SweeD (v3.2.1) [79]. The top 1% of likelihood values were considered to identify potential selective sweeps within highly differentiated genomic regions. The analysis first involved partitioning the population and chromosomes. Chromosome lengths were obtained from the VCF file header, and scanning window sizes were set accordingly. Next, we determined the length of each chromosome and set the size of the scanning window. To ensure uniform segmentation, we divided each chromosome into 10 kb grids, 100 grids per megabase. SweeD was then executed, and the results were visualized following generation of the output files. We performed Population Branch Statistic (PBS) analysis using PBScan (https://github.com/thamala/PBScan) [80] to detect signatures of selective sweeps among the Munage, other cultivated grapes, and ME wild grapes. The PBS output was ranked in descending order, and the top 1% of values were designated as outliers for subsequent Gene Ontology (GO) enrichment analysis.

### Transcriptome differential gene analysis

In this study, we analyzed RNA-seq data from three WM leaves (control), three WM peels, and nine RM peels, using WM2 as the reference genome for differential expression analysis. We first constructed an index of the haplotype reference genome with HISAT2’s `hisat2-build` (v.2.2.1) [65]. The transcriptome data were then aligned to the reference genome using HISAT2. The resulting SAM files were converted to BAM format, sorted, and indexed with SAMtools. In parallel, the gene annotation file in GFF3 format was converted to GTF format using `gffread` (v.0.12.7) [81]. Next, we employed `featureCounts` [82] to quantify gene expression by comparing the genomic positions of annotated features with the alignment positions of sequencing reads or fragments, thereby generating a gene expression matrix.

Subsequently, we applied DESeq2 [83] to estimate differential fold changes based on the gene expression matrix, obtaining the differential fold changes and significance *p*-values for each gene. Genes were filtered using the criteria |log2FC| ≥ 2 and padj <0.01, with upregulated and downregulated genes classified as ‘up’ and ‘down’, respectively.

## Supplementary Material

Web_Material_uhaf274
