# Supplementary material for: Haplotype-resolved assemblies provide insights into genomic makeup of the oldest grapevine cultivar (Munage) in China
Source: Hortic Res. 2025 Oct 20;13(1):uhaf274. doi: 10.1093/hr/uhaf274 (PMC12881859; doi:10.1093/hr/uhaf274)
Supplement: Web_Material_uhaf274 [file web_material_uhaf274.zip › TableS1-4.docx]

**Table S1. Comparison of chromosome length WM1, WM2, RM1 and RM2 assembly.**

| **Chr_ID** | **WM1_Length (bp)** | | **WM2_Length(bp)** | **RM1_Length(bp)** | **RM2_Length(bp)** |
| --- | --- | --- | --- | --- | --- |
| Chr1 | | 24828091 | 24323354 | 24400844 | 24715939 |
| Chr2 | | 21453684 | 21614472 | 21458218 | 21494222 |
| Chr3 | | 21956811 | 21593469 | 21536987 | 22006528 |
| Chr4 | | 27128911 | 26331393 | 26303377 | 27148028 |
| Chr5 | | 27039085 | 27554711 | 26969450 | 27557495 |
| Chr6 | | 25111505 | 24986057 | 25008310 | 25052721 |
| Chr7 | | 31173380 | 30529185 | 31731389 | 30587366 |
| Chr8 | | 24311910 | 25340795 | 25762510 | 24158373 |
| Chr9 | | 23234080 | 26743558 | 24817437 | 23004635 |
| Chr10 | | 26367376 | 27294045 | 25852394 | 26206440 |
| Chr11 | | 22244874 | 19954744 | 20024093 | 20402234 |
| Chr12 | | 23293471 | 25185014 | 24829081 | 23249738 |
| Chr13 | | 29582808 | 28442042 | 29828677 | 28352613 |
| Chr14 | | 29639168 | 28831715 | 29636154 | 28817980 |
| Chr15 | | 22030944 | 22111255 | 21856749 | 22288342 |
| Chr16 | | 24572247 | 23339235 | 23522504 | 24469924 |
| Chr17 | | 20749501 | 20463297 | 20156923 | 19676922 |
| Chr18 | | 37681528 | 37105468 | 37733526 | 37078949 |
| Chr19 | | 25703744 | 27610568 | 28088058 | 24351175 |

**Table S2. Comparison of genomic features of WM1, WM2, RM1 and RM2 assemblies.**

|  | **WM1** | **WM2** | **RM1** | **RM2** |
| --- | --- | --- | --- | --- |
| Total sequence length (bp) | 488103118 | 489354377 | 489516681 | 480619624 |
| Number of chromosomes | 19 | 19 | 19 | 19 |
| Contig N50 (Mb) | 24.32 | 26.33 | 15.55 | 17.08 |
| Annotated centromere | 19 | 19 | 19 | 19 |
| Annotated telomere | 36 | 36 | 34 | 34 |
| The number of gene | 33942 | 34034 | 35292 | 35007 |
| Repeat content (%) | 67.04% | 67.15% | 67.02% | 66.56% |
| BUSCO | 98.3% | 98.6% | 98.3% | 98.5% |

**Table S3. Gene Ontology (GO) analysis enrichment analysis of PN40024-specific homologous genes.**

**Table S4. Gene Ontology (GO) analysis enrichment analysis of WM1-specific homologous genes.**

**Table S5. The functional annotation of the unique orthologous genes in the WM2 genome.**

**Table S6. Gene Ontology (GO) analysis enrichment analysis of RM1-specific homologous genes.**

**Table S7. Gene Ontology (GO) analysis enrichment analysis of RM2-specific homologous genes.**

**Table S8. Grape samples used in the analysis:** Samples numbered 1 to 50 are the whole genome sequencing data obtained in previous studies, and others are the data obtained in this study.

**Table S9. Gene Ontology (GO) analysis enrichment analysis of the top 1% regions corresponding to Munage's Sweed.**

**Table S10. Gene Ontology (GO) analysis enrichment analysis of the top 1% regions as identified by PBS.**

**Table S11. Gene Ontology (GO) analysis enrichment analysis of the top 1% regions corresponding to WM's Sweed.**

**Table S12. Gene Ontology (GO) analysis enrichment analysis of the top 1% regions corresponding to RM's Sweed.**

**Table S13. Differential expression analysis: GO enrichment analysis of upregulated genes**

**Table S14. Differential expression analysis: GO enrichment analysis of downregulated genes**
